# *κ*-carrageenan oligosaccharides alleviate MDP induced rumen epithelial cell inflammatory damage by inhibiting the activation of NOD2/NF-κB pathway

**DOI:** 10.3389/fvets.2025.1626423

**Published:** 2025-07-09

**Authors:** Yimei Xiao, Xiaolin He, Jian Ma, Chunmei Du, Shangquan Gan, Fuquan Yin

**Affiliations:** ^1^College of Coastal Agriculture Science, Guangdong Ocean University, Zhanjiang, China; ^2^The Key Laboratory of Animal Resources and Breed Innovation in Western Guangdong Province, Department of Animal Science, Guangdong Ocean University, Zhanjiang, China

**Keywords:** κ-carrageenan oligosaccharides, inflammation, muramyl dipeptide, ruminal epithelial cells, NOD2/NF-κB signaling pathway

## Abstract

**Introduction:**

Under Subacute Ruminal Acidosis (SARA) conditions, harmful substances released by the massive lysis of ruminal bacteria are further degraded into small bacterial peptides, such as muramyl dipeptide (MDP). These degradation products are absorbed through the rumen wall and enter the bloodstream continuously, triggering a series of nutritional metabolic diseases and systemic pro-inflammatory responses. Therefore, inhibiting MDP-induced damage emerges as a novel target for preventing and alleviating SARA. Nutritional regulation serves as a critical strategy for enhancing the body’s resistance to inflammatory challenges. Moreover, plant-derived oligosaccharide extracts offer a promising approach for the prevention and control of animal diseases.

**Methods:**

In this study, the protective effects and possible molecular mechanisms of *κ*-carrageenan oligosaccharides (KOS) against MDP-induced damage in ovine ruminal epithelial cells (ORECs) were evaluated. The CCK8 assay, western blot analysis, ELISA, and PCR were employed in this study.

**Results:**

The results demonstrated that exposing ORECs to 25 μg/mL MDP for 6 h induced inflammatory damage. In contrast, pretreatment of ORECs with 75 μg/mL KOS for 9 h significantly enhanced cell viability, downregulated pro-inflammatory cytokine levels, restored immunoglobulin concentrations, reduced apoptosis rates, and regulated the expression of apoptosis-related genes under MDP stimulation. KOS exerted anti-inflammatory effects by scavenging ROS, improving tight junction barrier function, and inhibiting activation of the NOD2/NF-κB signaling pathway.

**Conclusion:**

Pretreatment with 75 μg/mL KOS for 9 h effectively alleviated MDP-induced inflammatory damage in ORECs by inhibiting the activation of the NOD2/NF-*κ*B pathway.

## 1 Introduction

Large-scale high-concentrate feeding has become the foundation of modern beef and sheep production. Nevertheless, this intensive feeding approach renders animals susceptible to acute or subacute ruminal acidosis (SARA), which disrupts ruminal metabolism, impairs productivity, and threatens animal health (1, 2). During SARA, a large number of ruminal bacteria die and disintegrate, resulting in the release of various harmful substances (3), including lipopolysaccharides (LPS), lipoteichoic acid (LTA), peptidoglycan (PGN), and muramyl dipeptide (MDP) (4). Once these bacterial products are absorbed through the ruminal wall and enter the bloodstream, they are likely to trigger nutritional metabolic disorders (5) and systemic pro-inflammatory responses (6, 7). MDP, one of the degradation products of PGN, is the smallest structural unit of the cell walls of both Gram-positive and Gram-negative bacteria (8). Almost all bacteria produce MDP during proliferation and lysis. However, while current research on ruminant inflammatory damage mainly centers on LPS and LTA, studies on MDP remain scarce (9). Bougarn et al. (10) found that MDP treatment significantly increased the gene expression of *TNF-α* in bovine mammary epithelial cells. Similarly, MDP can activate the transcription factor *NF-κB* and enhance the gene expression of *IL-8* in Caco2 cells (11). Moreover, Sun et al. (12) found that SB203580, a specific inhibitor of p38 MAPK, effectively downregulated the expression of pro-inflammatory cytokines TNF-*α* and IL-15 induced by MDP. These findings suggest that blocking MDP-induced damage represents a promising new target for preventing and alleviating SARA.

Nutritional regulation serves as a pivotal approach to modulate the body’s resistance against inflammatory challenges. As degradation products of carrageenan, carrageenan oligosaccharides are water-soluble linear sulfated oligosaccharides, formed by the alternating linkage of galactose and anhydrogalactose units via *α*-1,3- and *β*-1,4- glycosidic bonds (13). These oligosaccharides can be categorized into different types (e.g., *κ*-, *ι*-, *λ*-, and *γ*-) based on the number and position of sulfate group substitutions on the main-chain sugar rings (14). They display a wide range of remarkable functions, including anti-inflammatory, antioxidant, anti-tumor, and anti-pathogenic microbial activities (13, 15). Studies have shown that carrageenan oligosaccharides with lower molecular weight, higher degree of polymerization, and more sulfate groups tend to exhibit enhanced biological activities (16–18). Notably, the anti-inflammatory mechanisms of carrageenan oligosaccharides differ significantly across various cell types. Research has reported that *κ*-carrageenan oligosaccharides (KOS) can competitively bind to CD14 against LPS, thereby inhibiting the CD14/REL-dependent NF-κB pathway and reducing the expression of TNF-*α*, IL-1β, IL-8, COX-2, and other cytokines in RAW264.7 macrophages, which demonstrates significant anti-inflammatory activity (19). Yao et al. (20) found that KOS can inhibit LPS-activated microglia via the TLR4/NF-κB and p38/JNK MAPKs pathways, thereby suppressing the release of inflammatory cytokines and oxidative stress responses. Moreover, KOS can induce cellular autophagy through the AMPK/ULK1 pathway, regulating the immune response of microglia (21). However, it remains uncertain whether KOS can modulate MDP-induced inflammatory damage in ovine ruminal epithelial cells (ORECs). Therefore, the aim of this study was to analyze the effects of KOS on MDP-induced inflammatory damage in ORECs and explore the underlying mechanisms, thereby providing a theoretical basis for the application of KOS as a potential green feed additive in ruminant production.

## 2 Materials and methods

### 2.1 Reagents and drugs

KOS (Catalog No.: S231113KC1-3 K; purity > 90%; average molecular mass of 1.48 kDa) was purchased from Qingdao BZ Oligo Biotech Co., Ltd. (Qingdao, China). A stock solution of 500 μg/mL was prepared using complete medium, then serially diluted to different concentrations with complete medium as needed. The solution was stored at 4°C and used within 1 week. MDP (Catalog No.: GA20623; purity > 98%; molecular formula C_19_H_32_N_4_O_11_) was provided by GlpBio Technology (Montclair, California, USA). A 25 mg/mL stock solution was prepared with PBS and stored at −80°C, and subsequently diluted to various concentrations with complete medium before use. GSK717 (Catalog No.: GC60887; purity > 99.50%; molecular formula C_28_H_28_N_4_O_2_), also provided by GlpBio Technology (Montclair, California, USA), was used to prepare a 50 mM stock solution with DMSO and stored at −80°C. Before use, it was diluted to 5 μM with complete medium (the final concentration of DMSO was 0.01%). The complete medium consisted of 10% fetal bovine serum, 2% penicillin–streptomycin mixture, and 88% DMEM/F12 cell culture medium. The chemical reagents and detection kits used in this study are listed in Supplementary Table 2.

### 2.2 Cell culture

The ovine ruminal epithelial cells used in this study were provided by iCell Bioscience Inc. (Shanghai, China) and identified by immunofluorescence (Supplementary Figure 3). The cells were cultured in complete medium and routinely maintained in a cell culture incubator (ESCO Celmate, Singapore) at 37°C with 5% carbon dioxide (22).

### 2.3 Treatments

For the purposes of this study, ORECs were divided into the following treatment groups: (1) CON group: no treatment; (2) Cells were treated with different concentrations (0, 2, 10, 25, and 50 μg/mL) of MDP for 3, 6, 12, and 24 h; (3) MDP group: based on the screening results of MDP, cells were treated with 25 μg/mL of MDP for 6 h; (4) Cells were treated with different concentrations (0, 25, 50, 75, 100, and 150 μg/mL) of KOS for 3, 6, 9, 12, and 18 h; (5) Based on the time-screening results of KOS, cells were treated with KOS at concentrations of 0, 25, 50, 75, 100, and 150 μg/mL for 9 h, and then exposed to 25 μg/mL of MDP for 6 h; (6) KOS group: according to the screening results of the protective dose of KOS, cells were treated with 75 μg/mL of KOS for 9 h; (7) KOS + MDP group: cells were treated with 75 μg/mL of KOS for 9 h, and then exposed to 25 μg/mL of MDP for 6 h; (8) G + MDP group: cells were incubated with 5 μM of GSK717 for 1 h, and then exposed to 25 μg/mL of MDP for 6 h.

### 2.4 CCK-8

A 100 μL cell suspension at a density of 5,000 cells/mL was seeded into each well of a 96-well plate and cultured until cellular adhesion occurred. After various treatments, the old medium was discarded, and the cells were washed once with PBS. Then, 100 μL of CCK-8 incubation solution, prepared by mixing 10% Cell Counting Kit and 90% DMEM/F12 cell culture medium, was added to each well. The plates were then incubated at 37°C for 4 h. Subsequently, the absorbance at 450 nm was measured using an automatic microplate reader (BioTek, Vermont, USA), and cell viability was calculated according to the instructions of the CCK-8 detection kit (23). The experiment was performed across three independent biological replicates, each consisting of five technical replicates. Data were analyzed using the mean values of the technical replicates within each biological replicate.

### 2.5 Enzyme-linked immunosorbent assay (ELISA)

Cells were seeded into 6-well plates at a density of 8.0 × 10^5^ cells per well and cultured until cellular adhesion occurred. After different treatments, cells were scraped off completely using a cell scraper and transferred to enzyme-free centrifuge tubes. Subsequently, the cells were disrupted by an ultrasonic cell disruptor (Ningbo Sjialab Equipment Co., Ltd., Ningbo, China), centrifuged at 3000 r/min for 10 min at 4°C. The supernatants were collected and processed according to the instructions provided by the ELISA kit. Finally, the absorbance was measured with an automatic microplate reader (BioTek, Vermont, USA) (22, 23). The experiment was performed across three independent biological replicates, each consisting of three technical replicates. Data were analyzed using the mean values of the technical replicates within each biological replicate.

### 2.6 Reactive oxygen species (ROS)

Dichlorofuorescin diacetate (DCFH-DA) was employed to observe the intracellular levels of ROS. After various treatments, the spent medium was discarded, and the cells were washed once with PBS. Then, 2 mL of 5 μmol/L DCFH-DA incubation solution was added, and the cells were incubated in the dark at 37°C for 20 min. Subsequently, the incubation solution was removed, and the cells were gently washed five times with PBS. Finally, the cells were observed and photographed using an inverted fluorescence microscope (Olympus Corporation, Tokyo, Japan) (22). The experiment was performed across three independent biological replicates, and the average values were selected for analysis.

### 2.7 Flow cytometry detection of cell apoptosis

Cells were seeded into 6-well plates at a density of 8.0 × 10^5^ cells per well and cultured until cellular adhesion occurred. Following various treatments, cell apoptosis was detected using the Annexin V-FITC/PI method, with the detection steps performed according to the manufacturer’s instructions (22). The experiment was performed across three independent biological replicates, and the average values were selected for analysis.

### 2.8 Quantitative real-time PCR (qRT-PCR)

Following diferent treatments, total RNA was extracted using the FastPure Complex Tissue/Cell Total RNA Isolation Kit and reverse-transcribed into cDNA according to the instructions provided by the HisyGo RT Red SuperMix. The real-time PCR assay was conducted using a Bio-Rad real-time PCR system (Bio-Rad Laboratories, Inc., Hercules, California, USA) in a total reaction volume of 20 μL. All reactions were performed with an initial denaturation step at 95°C for 2 min, followed by 40 cycles of denaturation at 95°C for 15 s and combined annealing/extension at 62°C for 30 s. Subsequently, melt curves were investigated to identify PCR specificity. The experiment was performed across three independent biological replicates, each consisting of five technical replicates. Data were analyzed using the mean values of the technical replicates within each biological replicate. Relative mRNA expression levels were calculated using the 2^–ΔΔCt^ method (23). All primers, including the reference gene GAPDH, are synthesized by Shenggong Biotechnology Co., Ltd. (Shanghai, China) and listed in Supplementary Table 3.

### 2.9 Western blotting

Total cellular proteins were extracted from samples obtained in 4 independent biological experiments using PIRA lysis buffer. Protein quantification was performed with a BCA Protein Assay Kit following the manufacturer’s instructions strictly. Samples were stored at −80°C for long-term preservation. Equal amounts of denatured proteins separated by SDS-PAGE were transferred onto polyvinylidene fluoride (PVDF) membranes. Subsequently, the membranes blocked with TBST were incubated with the primary antibodies overnight at 4°C. After washing, the membranes were incubated with the secondary antibodies at room temperature for 1 h. Finally, after image development using an automatic chemiluminescence image analysis system (Tianneng Group, Shanghai, China), the target protein bands were evaluated using Gel-pro32 grayscale software (Media Cybernetics, Rockville, Maryland, USA) (22). In this study, *β*-actin was simultaneously tested as an internal reference. Information regarding the antibodies used in the Western blot analysis is provided in Supplementary Table 4.

### 2.10 Statistical analysis

After preliminary data organization in Excel, data analysis was conducted using SPSS 25.0 statistical software (SPSS Inc., Chicago, IL, USA). One-way ANOVA and Duncan’s multiple comparison tests were performed to determine the significance of differences between means. All analysis results were expressed as the mean ± standard deviation (SD), where *p* < 0.05 was considered statistically significant. Finally, GraphPad Prism 8.0.2 (California, USA) was used for figure plotting.

## 3 Results

### 3.1 Establishment of ORECs inflammatory injury model

**Figure 1:**
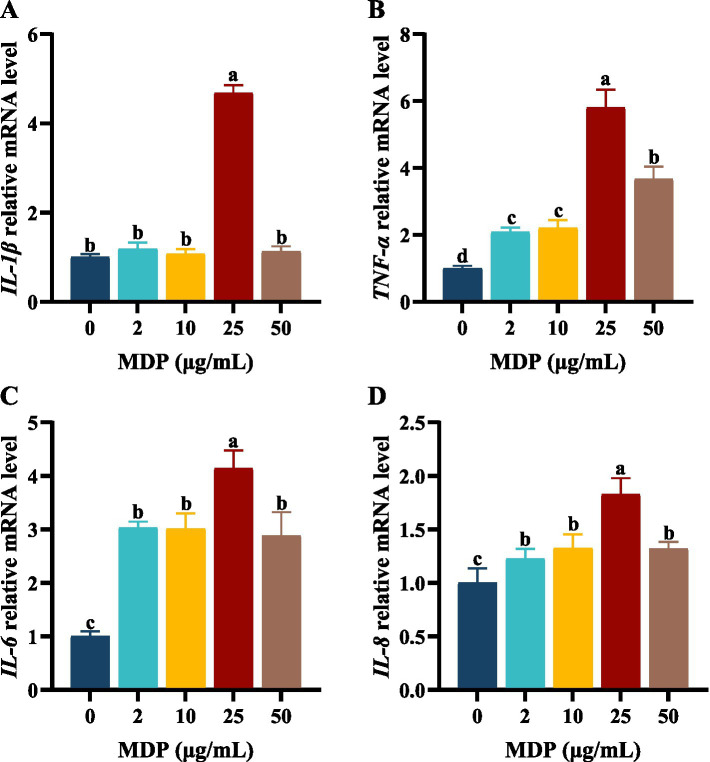
The effects of different MDP treatment concentrations on mRNA expression of inflammatory cytokine in ORECs. ORECs, ovine ruminal epithelial cells; MDP, muramyl dipeptide. **(A–D)** Relative mRNA expression levels of *IL-1β*, *TNF-α*, *IL-6*, and *IL-8*, respectively. TNF-α, Tumor necrosis factor-alpha; IL-1β, Interleukin-1 beta; IL-6, Interleukin-6; IL-8, Interleukin-8. Results are presented as the Mean±SEM. ^a–d^Values in the same row with different letters are significantly different (*p* < 0.05).

The first step of this study was to screen the concentrations and durations of MDP exposure for subsequent research. The relative contents of inflammatory cytokines in cell supernatants and the mRNA expression levels of inflammatory cytokines in ORECs were used as evaluation criteria. The results are shown in Supplementary Table 1 and Figure 1. When ORECs were co-cultured with different concentrations of MDP for 3, 6, and 12 h, the relative contents of pro-inflammatory cytokines increased to different extents. However, compared with the control group, when the MDP treatment duration was 24 h, the relative contents of TNF-*α*, IL-6, and IL-8 increased significantly at all MDP treatment concentrations (*p* < 0.05). Additionally, when the treatment time was 6 h, the relative contents of TNF-α, IL-1β, IL-6, and IL-8 in the MDP-10, MDP-25, and MDP-50 groups were significantly higher than those in the MDP-0 group, and the MDP-25 group showed the highest levels (*p* < 0.05). Therefore, based on the detection results of inflammatory cytokine contents, an exposure time of 6 h to MDP was selected for subsequent studies.

The effects of MDP concentrations ranging from 0 to 50 μg/mL on the mRNA expression levels of inflammatory cytokines are shown in Figure 1. When ORECs were treated with 25 μg/mL MDP for 6 h, the relative mRNA expression levels of inflammatory cytokines *IL-1β*, *TNF-α*, *IL-6*, and *IL-8* were the highest, all of which were significantly higher than those in other concentration treatment groups (*p* < 0.05). The mRNA expression levels of IL-6 and IL-8 in MDP-2, MDP-10, and MDP-50 groups were significantly higher than those in MDP-0 group (*p* < 0.05). In addition, the mRNA expression levels of TNF-α in MDP-2 and MDP-10 groups were significantly higher than those in MDP-0 group, but lower than those in MDP-50 group (*p* < 0.05). Therefore, a concentration of 25 μg/mL MDP was used in subsequent experiments.

### 3.2 Effect of KOS on the viability of ORECs

Cells were treated with KOS at concentrations ranging from 0 to 150 μg/mL for 3, 6, 9, 12, and 18 h. The optimal treatment time of KOS on ORECs was evaluated by the CCK-8 assay, and the results are shown in Table 1. After 6 h of culture, compared with the KOS-0 group, the cell viability of the KOS-50, KOS-75, and KOS-100 groups increased significantly (*p* < 0.05). After 9 h of culture, the cell viability of all treatment groups increased significantly compared with the KOS-0 group (*p* < 0.05). Notably, the cell viability of the KOS-75 group was significantly higher than that of the other groups (*p* < 0.05). After 12 h of culture, the cell viability of the KOS-100 and KOS-150 groups were significantly lower than those of the KOS-50 and KOS-75 groups (*p* < 0.05). After 18 h of culture, the cell viability of the KOS-150 group was significantly lower than that of the control group (*p* < 0.05). There were no significant differences among the remaining treatment groups. Therefore, based on the effect of KOS on the viability of ORECs, a pretreatment duration of 9 h with KOS was selected for subsequent studies.

**Table 1:** The effect of different concentrations of KOS and different treatment times on the activity of ORECs (%).

KOS	Time (h)					
(μg/mL)	3	6	9	12	18	*p*-value
0	100.00 ± 3.74	100.00 ± 2.23^c^	100.00 ± 1.74^e^	100.00 ± 3.37^bcd^	100.00 ± 3.28^a^	1.000
25	102.28 ± 5.06	103.03 ± 1.46^bc^	108.87 ± 1.49^c^	104.14 ± 2.89^abc^	99.79 ± 4.59^a^	0.084
50	106.53 ± 4.62^AB^	104.54 ± 1.50^BCb^	112.82 ± 1.99^Ab^	106.83 ± 3.75^ABab^	98.56 ± 5.15^Ca^	0.011
75	104.71 ± 4.90^BC^	108.33 ± 1.46^Ba^	121.17 ± 1.30^Aa^	108.90 ± 4.80^Ba^	98.05 ± 5.23^Ca^	<0.001
100	106.88 ± 2.69^A^	105.05 ± 1.98^Aab^	106.87 ± 1.52^Ac^	97.39 ± 3.89^Bcd^	92.83 ± 3.12^Ba^	<0.001
150	103.03 ± 4.07^A^	102.87 ± 2.17^Abc^	103.68 ± 1.84^Ad^	95.47 ± 3.79^Bd^	85.05 ± 2.20^Cb^	<0.001
*P*-value	0.383	0.003	<0.001	0.005	0.005	

ORECs, ovine ruminal epithelial cells; KOS, κ-carrageenan oligosaccharides; MDP, muramyl dipeptide. Results are presented as the Mean±SEM. In the same row^(A–C)^ or column^(a–e)^, the absence of letters or the presence of the same letters in superscripts is considered to indicate no significant difference (*p* > 0.05), while different letters are considered to indicate a significant difference (*p* < 0.05).

**Figure 2:**
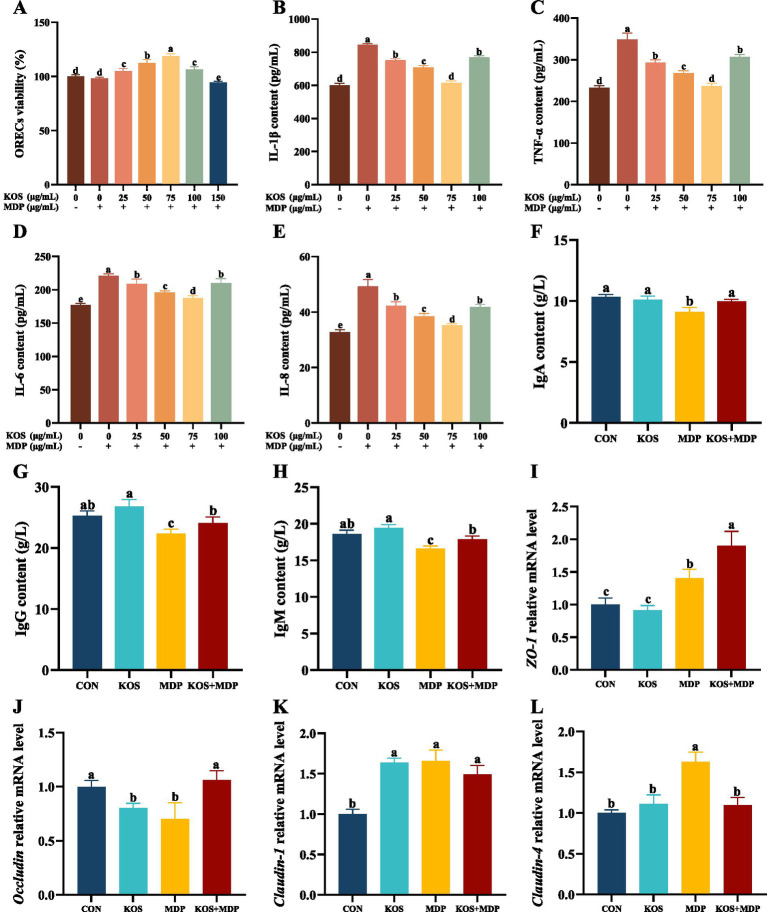
Effects of KOS on inflammatory factors, immunoglobulins, and tight junction proteins in ORECs induced by MDP. ORECs, ovine ruminal epithelial cells; KOS, κ-carrageenan oligosaccharides; MDP, muramyl dipeptide. CON group, cells were cultured only with complete medium; KOS group, cells were treated with 75 μg/mL KOS for 9 h; MDP group, cells were treated with 25 μg/mL MDP for 6 h; KOS + MDP group, cells were treated with 75 μg/mL KOS for 9 h followed by 25 μg/mL MDP for 6 h. **(A)** ORECs vitality test results. **(B–E)** The levels of IL-1β, TNF-α, IL-6, and IL-8, respectively. **(F–H)** The levels of IgA, IgG, and IgM, respectively. **(I–L)** Relative expression levels of *ZO-1*, *Occludin*, *Claudin-1*, and *Claudin-4*, respectively. Results are presented as the Mean±SEM. ^a–e^Values in the same row with different letters are significantly different (*p <* 0.05).

The effects of different concentrations of KOS with a 9 h pretreatment time on the viability of ORECs stimulated by MDP were further investigated, and the results are shown in Figure 2A. Compared with the CON group, exposure to MDP had no significant effect on the viability of ORECs. In contrast, compared with both the CON and MDP groups, the groups pretreated with 25, 50, 75, and 100 μg/mL KOS significantly increased the viability of ORECs (*p* < 0.05). Notably, cell viability was highest when the KOS concentration was 75 μg/mL (*p* < 0.05). However, when the pretreatment concentration of KOS was 150 μg/mL, cell viability was inhibited (*p* < 0.05), indicating that high concentrations of KOS could have a detrimental effect on ORECs when MDP is present.

### 3.3 Effects of KOS on inflammatory factors, immunoglobulins, and tight junction proteins in ORECs induced by MDP

To determine the optimal concentration of KOS, the effects of KOS pretreatment at concentrations ranging from 0 to 100 μg/mL on the cytokine levels in ORECs stimulated by MDP were further investigated. The results are shown in Figure 2B–E. Compared with the CON group, exposure to MDP significantly increased the levels of cellular inflammatory cytokines IL-1β, TNF-*α*, IL-6, and IL-8 (*p* < 0.05). In contrast, compared with the MDP group, the groups pretreated with 25, 50, 75, and 100 μg/mL KOS significantly decreased the levels of IL-1β, TNF-α, IL-6, and IL-8 (*p* < 0.05). Notably, the most effective reduction was observed when the KOS concentration was 75 μg/mL (*p* < 0.05). Therefore, 75 μg/mL was selected as the treatment concentration of KOS for subsequent experiments in this study.

The effects of KOS on the immunoglobulin contents in ORECs induced by MDP are presented in Figure 2F–H. Compared with the CON group, MDP stimulation significantly reduced the contents of IgA, IgG, and IgM in ORECs (*p* < 0.05). In contrast, compared with the MDP group, the contents of IgA, IgG, and IgM in the KOS + MDP group increased significantly (*p* < 0.05). However, the contents of IgG and IgM in the KOS + MDP group were significantly lower than those in the KOS group (*p* < 0.05).

As shown in Figure 2I–L, compared with the CON group, the *ZO-1* gene expression was significantly increased in the MDP and KOS + MDP groups (*p* < 0.05), with the highest expression in the KOS + MDP group. In addition, the gene expression of *Occludin* in the KOS and MDP groups was significantly lower than that in the CON and KOS + MDP groups (*p* < 0.05). The level of *Claudin-1* in the KOS, MDP, and KOS + MDP groups was significantly higher than that in the CON group (*p* < 0.05). The expression of *Claudin-4* in the MDP group was significantly higher than that in the other groups (*p* < 0.05).

### 3.4 Effects of KOS on the ROS content in ORECs induced by MDP

**Figure 3:**
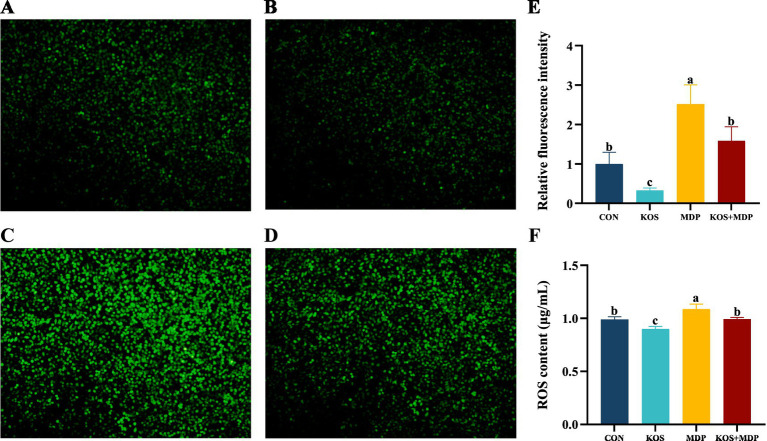
The effect of KOS on ROS content in ORECs induced by MDP (Scale = 20 μm). ORECs, ovine ruminal epithelial cells; KOS, κ-carrageenan oligosaccharides; MDP, muramyl dipeptide. CON group, cells were cultured only with complete medium; KOS group, cells were treated with 75 μg/mL KOS for 9 h; MDP group, cells were treated with 25 μg/mL MDP for 6 h; KOS + MDP group, cells were treated with 75 μg/mL KOS for 9 h followed by 25 μg/mL MDP for 6 h. **(A–D)** Fluorescence diagram of ROS in CON, KOS, MDP, and KOS + MDP groups, respectively. **(E)** Relative fluorescence intensity of ROS in each group. **(F)** ELISA detection results of ROS content in each group. Results are presented as the Mean±SEM. ^a–c^Values in the same row with different letters are significantly different (*p* < 0.05).

Figure 3 illustrates the detection results of ROS fluorescence intensity and content in each group, which are consistent. Compared with the CON group, the ROS content in the MDP group increased significantly (*p* < 0.05), while that in the KOS group decreased significantly (*p* < 0.05).

### 3.5 Effects of KOS on the apoptosis rate and apoptosis-related genes in ORECs induced by MDP

**Figure 4:**
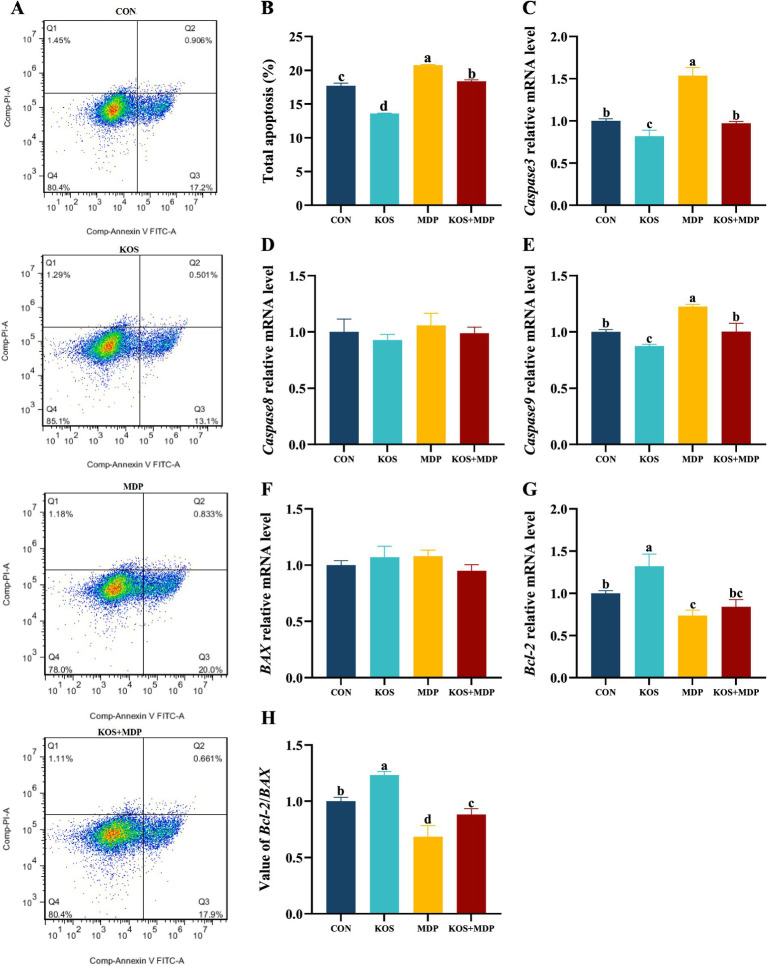
The effect of KOS on the apoptosis rate and apoptosis-related genes in ORECs induced by MDP. ORECs, ovine ruminal epithelial cells; KOS, κ-carrageenan oligosaccharides; MDP, muramyl dipeptide. CON group, cells were cultured only with complete medium; KOS group, cells were treated with 75 μg/mL KOS for 9 h; MDP group, cells were treated with 25 μg/mL MDP for 6 h; KOS + MDP group, cells were treated with 75 μg/mL KOS for 9 h followed by 25 μg/mL MDP for 6 h. **(A,B)** Apoptosis rate detection results of flow cytometry in each treatment group. **(C–G)** Relative expression levels of Caspase3, Caspase8, Caspase9, BAX, and Bcl-2, respectively. **(H)** Value of Bcl-2/BAX. Results are presented as the Mean±SEM. ^a–d^Values in the same row with different letters are significantly different (*p* < 0.05).

As shown in Figure 4A,B, the total apoptosis rate in the MDP group was significantly higher than that in the other three groups (*p* < 0.05). Compared with the CON group, the total apoptosis rate in the KOS group decreased significantly (*p* < 0.05), while that in the KOS + MDP group increased significantly (*p* < 0.05). To further investigate the effect of KOS on the apoptosis of MDP-induced damaged ORECs, the mRNA levels of apoptosis-related genes were detected, and the results are presented in Figure 4C–H. Compared with the CON group, the expression levels of *Caspase3* and *Caspase9* in the MDP group increased significantly (*p* < 0.05), while the levels of *Bcl-2* and the *Bcl-2*/*BAX* ratio decreased significantly (*p* < 0.05). The expression of Caspase3 and Caspase9 in the KOS group was significantly lower than those in the other groups (*p* < 0.05), whereas the expression of *Bcl-2* and the *Bcl-2*/*BAX* ratio were the highest (*p* < 0.05). The *Bcl-2*/*BAX* ratio in the KOS + MDP group was significantly lower than that in the CON and KOS groups (*p* < 0.05), but significantly higher than that in the MDP group (*p* < 0.05).

### 3.6 Effects of KOS on the NOD2/NF-κB pathway in ORECs induced by MDP

**Figure 5:**
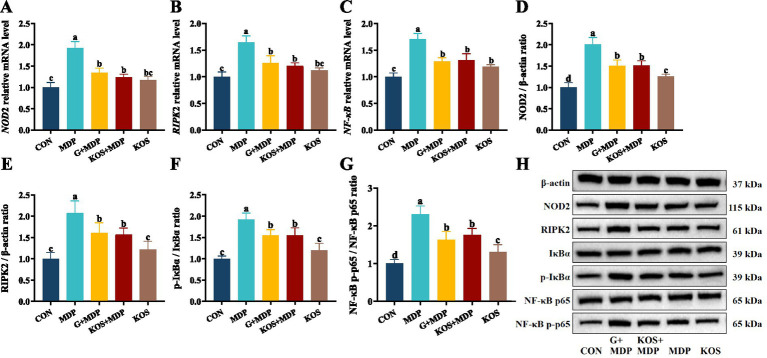
The effect of KOS on the expression levels of NOD2 and NF-κB pathway related genes and proteins in ORECs induced by MDP. ORECs, ovine ruminal epithelial cells; KOS, κ-carrageenan oligosaccharides; MDP, muramyl dipeptide; GSK717, inhibitor of NOD2 pathway. CON group, cells were cultured only with complete medium; KOS group, cells were treated with 75 μg/mL KOS for 9 h; MDP group, cells were treated with 25 μg/mL MDP for 6 h; KOS + MDP group, cells were treated with 75 μg/mL KOS for 9 h followed by 25 μg/mL MDP for 6 h; G + MDP group, cells were treated with 5 μM of GSK717 for 1 h followed by 25 μg/mL MDP for 6 h. **(A–C)** Relative expression levels of *NOD2*, *RIPK2*, and *NF-κB*, respectively. **(D–G)** The grayscale analysis results for NOD2, RIPK2, p-IκBα/IκBα and NF-κB p65/p-NF-κB p65, respectively. **(H)** The results of western blotting. Results are presented as the Mean±SEM. ^a–d^Values in the same row with different letters are significantly different (*p* < 0.05).

To further explore the mechanism by which KOS acts on MDP-induced inflammatory damage in ORECs, this study detected the gene expression levels of key signaling factors involved in MDP intracellular signal transduction, as well as the initiation and activation of inflammatory pathways. As shown in Figure 5A–C, compared with the CON group, the mRNA expression levels of *NOD2*, *RIPK2*, and *NF-κB* in the MDP, G + MDP and KOS + MDP groups increased significantly (*p* < 0.05), with the MDP group exhibiting the highest levels. In addition, compared with the CON group, the gene expression of *NF-κB* in the KOS group was significantly upregulated (*p* < 0.05).

Western blotting was used to measure the levels of proteins associated with the NOD2/NF-κB pathways. The effect of KOS on the expression of key proteins in the NF-κB signaling pathway of ORECs is shown in Figure 5D–H. The protein expression of NOD2 and RIPK2, as well as the phosphorylation levels of IκBα and NF-κB p65, was significantly higher in the MDP group than in the other groups (*p* < 0.05). Compared with the CON group, the expression of NOD2 and RIPK2, as well as the phosphorylation levels of IκBα and NF-κB p65, was significantly upregulated in the G + MDP and KOS + MDP groups (*p* < 0.05). The expression of NOD2 and RIPK2, along with the phosphorylation levels of IκBα and NF-κB p65, was significantly lower in the KOS group than in the G + MDP and KOS + MDP groups, but the expression of NOD2 and the phosphorylation level of NF-κB p65 were significantly higher than those in the CON group (*p* < 0.05).

## 4 Discussion

### 4.1 MDP induces inflammatory damage in ORECs

Inflammation can be triggered by various factors, including thrombosis, immune system disorders, cancer, infections, chemical exposure, physical injuries, or neurological diseases (24). Notably, many infections caused by viral, bacterial, fungal, and protozoan pathogens can lead to inflammation. Specifically, as the minimal structural unit of the cell walls of both Gram-positive and Gram-negative bacteria, MDP, upon entering the body, can induce the expression of cytokines, thereby activating and regulating the inflammatory response. During SARA, harmful substances generated by the massive disintegration of ruminal bacteria are further degraded into small bacterial peptides such as MDP, which continuously induce inflammatory damage in the ruminal epithelium. Therefore, it is highly necessary to establish an *in vitro* inflammatory damage model using MDP as the stimulant. In this study, ORECs were exposed to five different doses of MDP at a concentration gradient ranging from 0 to 50 μg/mL. The results indicated that increasing concentrations and incubation times did not have an adverse effect on the viability of ORECs but could enhance the relative contents of pro-inflammatory cytokines to varying extents. Specifically, after a 6 h treatment, the relative contents of TNF-*α*, IL-1β, IL-6, and IL-8 in the 10, 25, and 50 μg/mL treatment groups were significantly higher than those in the 0 and 2 μg/mL groups.

When the treatment duration was 6 h, the effects of different MDP treatment concentrations on the mRNA expression levels of inflammatory cytokines were further examined. The results demonstrated that as the MDP treatment concentration increased, the mRNA expression levels of pro-inflammatory factors first increased and then decreased. We speculate that this may be related to the transmembrane transport mechanism of MDP (25, 26). When the MDP treatment concentration was 25 μg/mL, the relative expressions of *IL-1β*, *TNF-α*, *IL-6*, and *IL-8* were significantly higher than those in other concentration treatment groups, indicating a strong pro-inflammatory response. Therefore, in this study, 25 μg/mL MDP with a stimulation time of 6 h was selected as the optimal condition for constructing an inflammatory damage model of ORECs *in vitro*.

### 4.2 KOS alleviates MDP induced inflammatory damage through NOD2/NF-κB pathway

As the degradation products of carrageenan, carrageenan oligosaccharides exhibit various biological functions, such as anti-inflammatory, antioxidant, antibacterial, and antitumor activities (13). At present, it is not clear whether KOS has an effect on sheep, so we first explored the effect of KOS on rumen epithelial cells. In this study, ORECs were pretreated with KOS at concentrations ranging from 0 to 150 μg/mL for 3, 6, 9, 12, and 18 h. The results showed that within a treatment time of 6 to 12 h, cell viability increased initially and then decreased with the increase in KOS pretreatment concentration, and the highest cell viability in each concentration group was observed at 9 h. Subsequently, cells were pretreated with varying concentrations of KOS for 9 h and then exposed to MDP. The results showed that cell viability reached the highest level when pretreated with 75 μg/mL KOS. Conversely, a significant decline in cell viability was observed upon increasing the KOS concentration to 150 μg/mL. These findings indicate that KOS exerts a biphasic effect on cell viability, suggesting that high concentrations of KOS may be toxic to ORECs. This is similar to the report by Qiu et al. (22) on the effect of alginate oligosaccharides on the activity of ORECs. This may be related to the imbalance of cell osmotic pressure caused by high concentrations of oligosaccharides. Additionally, this study found that the alleviating effect of KOS on MDP-induced inflammatory damage was related to the pretreatment concentration. The optimal resistance to inflammatory challenges was achieved at a KOS addition concentration of 75 μg/mL, which is consistent with the findings of Guo et al. (19). Immunoglobulins can maintain immune homeostasis through mucosal protection and systemic anti-inflammatory effects, and regulate immunity in autoimmune and inflammatory diseases (27). In this study, MDP stimulation significantly decreased the contents of IgA, IgG, and IgM in ORECs, while KOS pretreatment alleviated this negative impact. These findings suggest that KOS can mitigate MDP-induced inflammatory damage by regulating the expression of cytokines and immunoglobulins.

Tight junctions, as the most crucial component of the intercellular connections in ruminal epithelial cells, are responsible for preventing microbial invasion, halting the spread of toxins, and regulating the flux of water-soluble molecules between cells. The functional integrity of tight junctions is essential for maintaining the relative stability of the ruminal internal environment and exerting the barrier function of the epithelium (28). Previous studies have shown that MDP-induced cellular inflammatory damage is associated with NOD2-mediated signaling (29, 30). MDP exerts bidirectional regulatory effects on intestinal immunity and microbiota homeostasis under different physiological and pathological conditions through the NOD2 signaling pathway and inflammatory mediators (31). On the one hand, MDP activates the NOD2 signaling pathway, thereby promoting the secretion of antibacterial peptides, upregulating the expression of tight junction proteins, repairing the intestinal epithelium, and exerting an anti-colitis effect (32, 33). On the other hand, MDP activates NOD2, triggering the NF-κB signaling cascades, which promotes the release of inflammatory factors and subsequently induces inflammatory damage. Pro-inflammatory cytokines increase the permeability of intestinal epithelial tight junctions, disrupt the barrier function, and accelerate the progression of inflammation and diseases (28, 34). In this study, MDP stimulation significantly upregulated the mRNA expression levels of *ZO-1*, *Claudin-1*, and *Claudin-4* in ORECs, while downregulating *Occludin* expression. This phenomenon may be attributed to the bidirectional regulatory effect of MDP on the tight junctions barrier via the NOD2 receptor. In contrast, KOS pretreatment not only further increased *ZO-1* expression but also alleviated the MDP-induced downregulation of *Occludin*, potentially due to KOS’s regulatory effect on pro-inflammatory factors. Therefore, the improvement of ORECs barrier function may represent one of the mechanisms by which KOS exerts its anti-inflammatory effects.

ROS play a crucial role in the body’s immune system, contributing to the establishment of innate and adaptive immunity and maintaining internal environmental homeostasis (35, 36). On one hand, high levels of ROS can promote the degradation of IκB, activate the nuclear translocation of NF-κB, and upregulate the levels of pro-inflammatory mediators (37). On the other hand, during the acute inflammatory response, a large number of cytokines and inflammatory mediators are rapidly released, leading to increased tissue oxygen consumption and respiratory burst. Consequently, a large amount of ROS is generated within a short time, effectively eliminating pro-inflammatory factors (35). Previous studies have shown that MDP-induced NOD2 activation promotes the production of ROS (38). MDP translocation in the gut can activate the NOD2 signaling pathway, resulting in organ damage, inflammatory responses, and mitochondrial dysfunction in rats, with MDP levels positively correlated with IL-6 levels (39). These findings are consistent with the results of this study, in which MDP stimulation upregulated the levels of ROS and inflammatory factors. Notably, KOS exhibits significant scavenging and reducing abilities against oxidative free radicals, such as superoxide anions, DPPH, and hydroxyl radicals (40). This study found that KOS can significantly reduce the ROS content in ORECs, and KOS pretreatment can alleviate the increase in ROS levels caused by MDP, indicating that KOS has good antioxidant capacity. Therefore, the ability of KOS to scavenge ROS may be one of the mechanisms underlying its anti-inflammatory effects.

Inflammatory responses and apoptosis are intricately intertwined. The ability of ROS to induce cell apoptosis was confirmed as early as 1991 (41). This study found that KOS protection treatment reduced the total apoptosis rate of cells to a level close to that of the CON group. Langford et al. (42) found that local exposure of rabbit ocular surfaces to MDP triggered non-infectious exudative conjunctivitis, resulting in a significant upregulation of *Caspase3* and *NF-κB* levels in the conjunctiva and tear fluid. This observation indicates that MDP stimulation can trigger Caspase-mediated acute conjunctival epithelial lesions. In this study, KOS protection treatment effectively mitigated the negative effects of MDP, significantly reducing the expression levels of *Caspase3* and *Caspase9*. Meanwhile, it markedly increased the *Bcl-2*/*BAX* ratio. These findings suggest that KOS can protect ORECs from MDP-induced excessive apoptosis.

**Figure 6:**
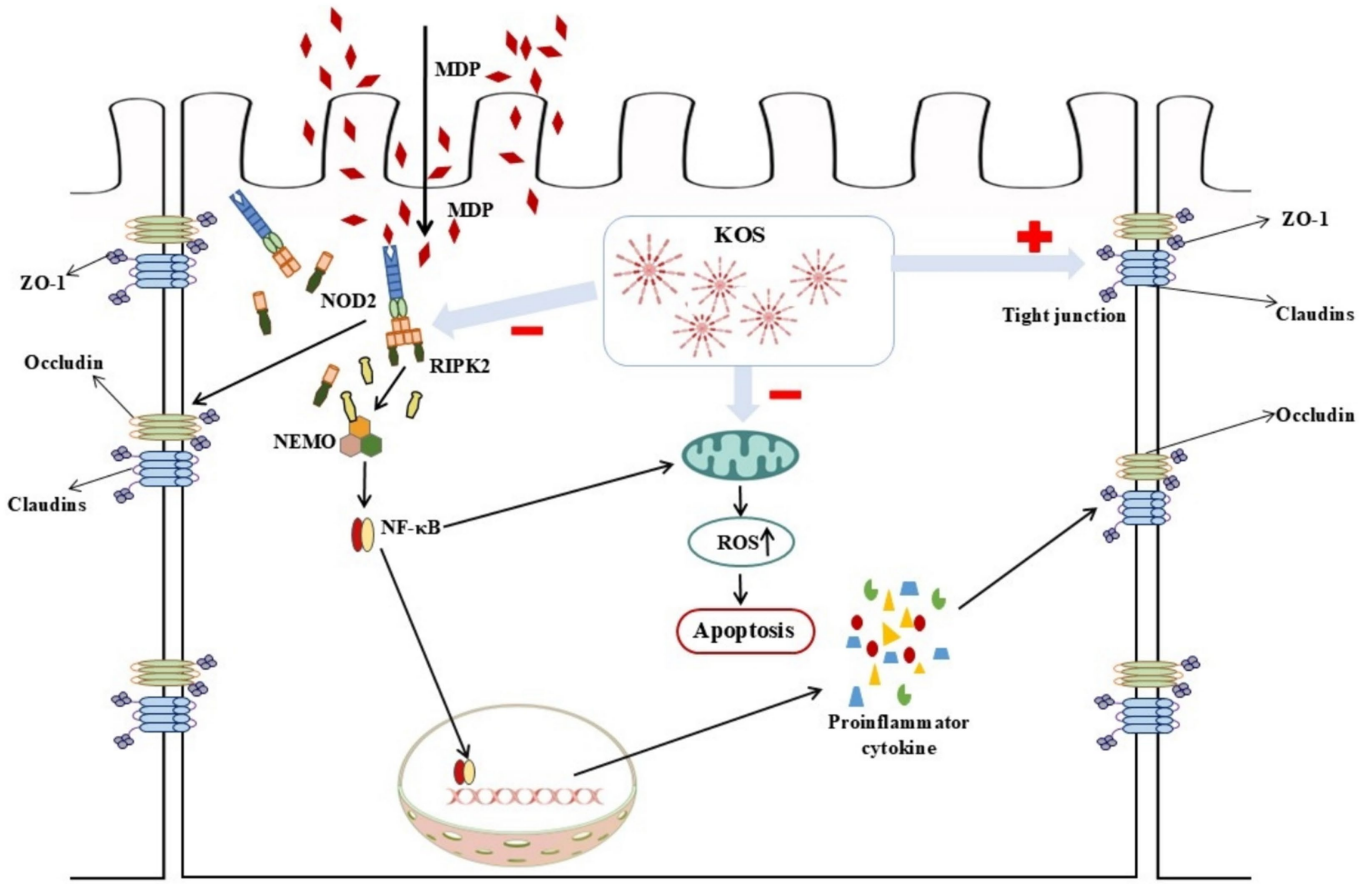
Schematic diagram illustrating how KOS can alleviate MDP-induced inflammatory damage by inhibiting the NOD2/NF-κB pathway.

A substantial amount of evidence currently has demonstrated that NOD2 is the direct receptor of MDP (43–46). Under normal conditions, NOD2 remains in an auto-inhibited inactive state (47). Once activation by MDP, the NOD2 receptor recruits and ubiquitinates RIPK2 through homotypic CARD domain interactions. Activated RIPK2, on the one hand, triggers the nuclear translocation of NF-κB, thereby upregulating the levels of pro-inflammatory mediators and promoting the secretion of antimicrobial peptides. On the other hand, it activates MAP kinases (such as p38, ERK, and JNK), which in turn activate the transcription factor AP-1 (48). NF-κB, a transcription factor regulating early gene expression, primarily functions as the p65-p50 heterodimer and promotes transcription of downstream inflammatory factors upon activation (49, 50). Consistent with previous findings, the results of this study indicate that MDP stimulation significantly activates the NOD2/NF-κB signaling pathway, which is further confirmed by Western blotting. Studies have shown that KOS inhibits inflammatory cytokine release via the NF-κB pathway and protects macrophages (19) and microglia (20) from damage induced by overactivation, consistent with the results of this study. GSK717, a potent and selective inhibitor of the NOD2 signaling pathway, competitively binds to the NOD2 receptor against MDP, suppressing NOD2 signaling without blocking other NF-κB induction pathways (51, 52). The results of this study revealed that incubating cells with 5 μM GSK717 for 1 h prior to MDP exposure effectively inhibited NOD2 signaling and downregulated the phosphorylation levels of IκBα and NF-κB p65. Notably, KOS pretreatment exerted effects similar to those of GSK717. These findings suggest that KOS may mitigate MDP-induced inflammatory injury by inhibiting NOD2 receptor signaling and subsequently suppressing the activation of downstream pathways. Certainly, as a unique digestive organ in ruminants, the rumen exhibits a complex physiological environment that differs significantly from *in vitro* epithelial cell models. Our study, which is limited to cell-based experiments, has inherent limitations. Additionally, the absence of *a priori* statistical power calculation represents another limitation. Future research should integrate animal trials to further investigate how KOS protects the ruminal epithelium against inflammatory damage. Additionally, unresolved questions that warrant further investigation include the differences in anti-inflammatory capacity between KOS and known agents (e.g., dexamethasone), whether KOS targets the NOD2 receptor, and whether KOS indirectly inhibits NF-κB pathway activation through other receptors (e.g., TLR4) or non-receptor mechanisms (e.g., antioxidant effects). A schematic diagram illustrating the effects of KOS on MDP-induced inflammatory damage in ORECs is shown in Figure 6.

## 5 Conclusion

In summary, the findings of this study indicate that exposure to 25 μg/mL MDP for 6 h can induce inflammatory damage in ORECs. In contrast, pretreatment with 75 μg/mL KOS for 9 h effectively alleviates MDP-induced inflammatory damage by inhibiting the activation of the NOD2/NF-κB pathway, indicating that KOS has the potential to prevent and alleviate SARA damage. Therefore, future research should integrate animal trials to further investigate the potential applications of KOS as a green feed additive.

## Data Availability

The datasets presented in this study can be found in online repositories. The names of the repository/repositories and accession number(s) can be found in the article/Supplementary material.
