# Supplementary material for: κ-carrageenan oligosaccharides alleviate MDP induced rumen epithelial cell inflammatory damage by inhibiting the activation of NOD2/NF-κB pathway
Source: Front Vet Sci. 2025 Jul 9;12:1626423. doi: 10.3389/fvets.2025.1626423 (PMC12285590; doi:10.3389/fvets.2025.1626423)
Supplement: Supplementary file 1 [file Table_1.docx]

Supplementary Material

# Supplementary Figures and Tables

**Supplementary Table 1.** The effects of different concentrations of MDP and different treatment times on the relative content of inflammatory cytokine in ORECs

| MDP | Time (h) | | | | |
| --- | --- | --- | --- | --- | --- |
| (μg/mL) | 3 | 6 | 12 | 24 | *P*-value |
| IL-1β |  |  |  |  |  |
| 0 | 1.000±0.025^c^ | 1.000±0.046^c^ | 1.000±0.026^b^ | 1.000±0.031 | 1.000 |
| 2 | 1.030±0.022^bc^ | 1.029±0.053^bc^ | 1.010±0.015^b^ | 1.027±0.024 | 0.853 |
| 10 | 1.071±0.013^Aab^ | 1.097±0.036^Ab^ | 1.076±0.025^Aa^ | 0.991±0.034^B^ | 0.009 |
| 25 | 1.102±0.035^Ba^ | 1.207±0.041^Aa^ | 1.110±0.038^Ba^ | 1.059±0.046^B^ | 0.011 |
| 50 | 1.047±0.009^Ab^ | 1.077±0.032^Ab^ | 1.086±0.032^Aa^ | 0.945±0.056^B^ | 0.005 |
| *P*-value | 0.003 | 0.001 | 0.003 | 0.052 |  |
| TNF-α |  |  |  |  |  |
| 0 | 1.000±0.031^c^ | 1.000±0.041^c^ | 1.000±0.022^c^ | 1.000±0.047^c^ | 1.000 |
| 2 | 1.013±0.020^bc^ | 1.022±0.042^c^ | 1.058±0.029^bc^ | 1.076±0.040^b^ | 0.155 |
| 10 | 1.046±0.033^bc^ | 1.102±0.039^b^ | 1.071±0.038^b^ | 1.083±0.051^ab^ | 0.454 |
| 25 | 1.116±0.028^Ba^ | 1.255±0.036^Aa^ | 1.157±0.042^Ba^ | 1.133±0.017^Bab^ | 0.003 |
| 50 | 1.069±0.044^ab^ | 1.092±0.029^b^ | 1.149±0.049^a^ | 1.149±0.022^a^ | 0.072 |
| *P*-value | 0.009 | <0.001 | 0.002 | 0.006 |  |
| IL-6 |  |  |  |  |  |
| 0 | 1.000±0.021^c^ | 1.000±0.004^e^ | 1.000±0.004^c^ | 1.000±0.018^d^ | 1.000 |
| 2 | 1.015±0.016^c^ | 1.039±0.008^d^ | 1.031±0.016^c^ | 1.035±0.011^c^ | 1.000 |
| 10 | 1.062±0.028^Ab^ | 1.130±0.023^Ab^ | 1.045±0.025^Bbc^ | 1.076±0.013^Bb^ | 0.001 |
| 25 | 1.171±0.019^Ba^ | 1.293±0.013^Aa^ | 1.131±0.039^Ca^ | 1.128±0.008^Ca^ | <0.001 |
| 50 | 1.097±0.028^b^ | 1.103±0.012^c^ | 1.077±0.030^b^ | 1.067±0.011^b^ | 0.121 |
| *P*-value | <0.001 | <0.001 | 0.001 | 0.006 |  |
| IL-8 |  |  |  |  |  |
| 0 | 1.000±0.057^d^ | 1.000±0.015^d^ | 1.000±0.021^c^ | 1.000±0.010^d^ | 1.000 |
| 2 | 1.210±0.041^Ac^ | 1.072±0.033^BCc^ | 1.112±0.037^Bb^ | 1.038±0.010^Cc^ | 0.001 |
| 10 | 1.328±0.024^Ab^ | 1.206±0.024^Bb^ | 1.247±0.029^Ba^ | 1.079±0.026^Cb^ | <0.001 |
| 25 | 1.400±0.031^Ba^ | 1.453±0.028^Aa^ | 1.281±0.022^Ca^ | 1.120±0.017^Da^ | <0.001 |
| 50 | 1.282±0.047^Ab^ | 1.172±0.027^Bb^ | 1.120±0.016^BCb^ | 1.069±0.031^Cbc^ | <0.001 |
| *P*-value | <0.001 | <0.001 | <0.001 | <0.001 |  |

^1^ORECs: ovine ruminal epithelial cells; MDP: muramyl dipeptide. Results are presented as the Mean±SEM. In the same row^(A-C)^ or column^(a-e)^, the absence of letters or the presence of the same letters in superscripts is considered to indicate no significant difference (*P*>0.05), while different letters are considered to indicate a significant difference (*P* < 0.05).

**Supplementary Table 2.** Main reagents and kit

| Reagents and kits | Item number/Batch | Manufacturing company | Headquarters address |
| --- | --- | --- | --- |
| Fetal bovine serum | Z7186FBS-500 | ZETA LIFE | San Francisco, California, USA |
| DMEM/F12 | BM0013 | ZETA LIFE | San Francisco, California, USA |
| Penicillin-Streptomycin mixture | S230824 | Serena life science technology development (Shanghai) CO., Ltd. | Shanghai, China |
| 0.25% Trypsin-EDTA | 2882461 | Gibco Life Technologies | GrandIsland, New York, USA |
| PBS | - | Gibco Life Technologies | GrandIsland, New York, USA |
| Sheep interleukin (IL-6) ELISA kit | YJ973652 | Shanghai Enzyme-linked Biotechnology Co., Ltd. | Shanghai, China |
| Sheep interleukin (IL-8) ELISA kit | YJ036680 | Shanghai Enzyme-linked Biotechnology Co., Ltd. | Shanghai, China |
| Sheep interleukin (IL-1β) ELISA kit | MM-012001 | Jiangsu Meimian Industrial Co. Ltd. | Yancheng, China |
| Sheep tumor necrosis factor alpha (TNF-α) ELISA kit | MM-3693801 | Jiangsu Meimian Industrial Co. Ltd. | Yancheng, China |
| FastPure Complex Tissue/Cell Total RNA Isolation Kit | RC113-01 | Vazyme Biotech Co.,Ltd | Nanjing, China |
| HisyGo RT Red SuperMix for qPCR (+gDNA Wiper) | 7E0710G4 | Vazyme Biotech Co.,Ltd | Nanjing, China |
| ChamQ Blue Universal SYBR qPCR Master Mix | 7E751K3 | Vazyme Biotech Co.,Ltd | Nanjing, China |
| Cell Counting Kit | K009-1000T | ZETA LIFE | San Francisco, California, USA |
| Sheep immunoglobulin A (Ig-A) ELISA kit | YJ025601 | Shanghai Enzyme-linked Biotechnology Co., Ltd. | Shanghai, China |
| Sheep immunoglobulin G (Ig-G) ELISA kit | YJ025602 | Shanghai Enzyme-linked Biotechnology Co., Ltd. | Shanghai, China |
| Sheep immunoglobulin M (Ig-M) ELISA kit | YJ002334 | Shanghai Enzyme-linked Biotechnology Co., Ltd. | Shanghai, China |
| Sheep Reactive Oxygen Species (ROS) ELISA Kit | MM-8110501 | Jiangsu Meimian Industrial Co. Ltd. | Yancheng, China |
| 2.5% trypsin (without EDTA and phenol red) | BL1096A | Biosharp | Hefei, China |
| Annexin V-FITC/PI Cell Apoptosis Detection Kit | A211-02 | Beyotime Biotechnology | Shanghai, China |
| ROS Assay Kit (DCFH-DA) | S0033S | Beyotime Biotechnology | Shanghai, China |
| RIPA lysis buffer and protease inhibitor | R0020 | Beijing Solarbio Science&Technology Co.,Ltd | Beijing, China |
| BCA Protein Concentration Determination Kit | P0011 | Beyotime Biotechnology | Shanghai, China |
| 5×Sample Buffer Solution | P0015L | Beyotime Biotechnology | Shanghai, China |
| 10×TBST | - | Jiangsu Aidisheng Biotechnology Co., Ltd | Yancheng, China |
| SDS-PAGE gel preparation kit | P1200 | Beijing Solarbio Science&Technology Co.,Ltd | Beijing, China |
| ColorMixed Protein Marker（11-180 KD） | PR1910 | Beijing Solarbio Science&Technology Co.,Ltd | Beijing, China |
| ECL Western Blotting Substrate | P0018M | Beyotime Biotechnology | Shanghai, China |
| Blotting Grade | P0216-1500g | Beyotime Biotechnology | Shanghai, China |
| Methanol | 80080418 | Sinopharm Chemical Reagent Co., Ltd | Shanghai, China |
| 10×Wet Transfer Buffer | - | Jiangsu Aidisheng Biotechnology Co., Ltd | Yancheng, China |
| 5×Tris-Glycine Running Buffer | - | Jiangsu Aidisheng Biotechnology Co., Ltd | Yancheng, China |
| MerckMillipore PVDF (0.45µm) | IPVH00010 | Merck Millipore | Darmstadt, Germany |
| MerckMillipore PVDF (0.2µm) | ISEQ00010 | Merck Millipore | Darmstadt, Germany |

**Supplementary Table 3.** Primer Sequence

| Genes | | GenBank ID | Primer sequence (5’-3’) | Length |
| --- | --- | --- | --- | --- |
| *GAPDH* | | XM_060411595.1 | F: GGCGTGAACCACGAGAAGTA | 141 |
|  |  |  | R: GGCGTGGACAGTGGTCATAA |  |
| *IL-1β* | NM_001009465.2 | | F: CAACCGTACCTGAACCCATCA | 105 |
|  |  |  | R: GTTGGGTGCAGCTCTTCATCT |  |
| *TNF-α* | NM_001024860.1 | | F: GCTGCACTTCGGGGTAATCG | 105 |
|  |  |  | R: GAGGCTTGAGAAGATGACCTGAG |  |
| *IL-6* | NM_001009392.1 | | F: ATCTGGGTTCAATCAGGCGAT | 89 |
|  |  |  | R: ACTCGTTCTGGAGGAAGTCCA |  |
| *IL-8* | KC912524.1 | | F: GCTGGCTGTTGCTCTCTTGGC | 127 |
|  |  |  | R: GGGGTGGAAAGGTGTGGAATGTG |  |
| *Claudin-1* | NM_001185016.1 | | F: CCCGGTCAATGCCAGGTATG | 113 |
|  |  |  | R: TTGTTTTCCGGGGACAGGAG |  |
| *Claudin-4* | [NM_001185017.2](https://www.ncbi.nlm.nih.gov/entrez/viewer.fcgi?db=nucleotide&id=1371543315) | | F: GAGCCCCAAGGCCAAGATTA | 99 |
|  |  |  | R: ATGACGTTGTTAGCCGTCCA |  |
| *Occludin* | [XM_060400238.1](https://www.ncbi.nlm.nih.gov/entrez/viewer.fcgi?db=nucleotide&id=2607037035) | | F: CCCAATGTGGAAGAGTGGGTTA | 140 |
|  |  |  | R: GGATACGGTCGCTTCTCGTT |  |
| *ZO-1* | [XM_060401409.1](https://www.ncbi.nlm.nih.gov/entrez/viewer.fcgi?db=nucleotide&id=2607041565) | | F: CAAGTTCCCGGCGTATGAGA | 149 |
|  |  |  | R: CCCGAGGTTCACTTTTTGCG |  |
| *BCL-2* | [XM_060405321.1](https://www.ncbi.nlm.nih.gov/entrez/viewer.fcgi?db=nucleotide&id=2607080396) | | F: GTCAGTGGGAACCTTTGCGA | 132 |
|  |  |  | R: CTCTGCACGCTGGTTGAAAG |  |
| *BAX* | [XM_027978594.3](https://www.ncbi.nlm.nih.gov/entrez/viewer.fcgi?db=nucleotide&id=2607029332) | | F: CAAACTGGTGCTCAAGGCCC | 87 |
|  |  |  | R: CGCTCTCGAAGGAAGTCCAAT |  |
| *Caspase3* | [XM_060406953.1](https://www.ncbi.nlm.nih.gov/entrez/viewer.fcgi?db=nucleotide&id=2607095548) | | F: GAACTTCCACGAAAATACTGGCAT | 84 |
|  |  |  | R: AAGCTCGTGAAGGTTTCCCT |  |
| *Caspase8* | [XM_042244960.2](https://www.ncbi.nlm.nih.gov/nucleotide/2607052333?report=gbwithparts) | | F: GCTGGATGATGACATGACTTTGCT | 97 |
|  |  |  | R: ACAGATTCTTTTCAGGGTGTCCA |  |
| *Caspase9* | [XM_060396599.1](https://www.ncbi.nlm.nih.gov/entrez/viewer.fcgi?db=nucleotide&id=2607009910) | | F: TGCAGGTTGCCTCGCTTT | 86 |
|  |  |  | R: TGAGCCTGCCCTCTGAATGT |  |
| *NOD2* | [XM_042231600.2](https://www.ncbi.nlm.nih.gov/nucleotide/2607021500?report=gbwithparts) | | F: ATCTTCACATCATCCCAGCGG | 146 |
|  |  |  | R: TACTTCTTACAGGCAGCATCTTCA |  |
| *RIPK2* | [XM_012184164.5](https://www.ncbi.nlm.nih.gov/nucleotide/2607277225?report=gbwithparts) | | F: CTGGTCCACAGGAGGAATCAT | 84 |
|  |  |  | R: GAGTTGACAGGGACACTGAGG |  |
| *NF-κB* | [XM_027960475.2](https://www.ncbi.nlm.nih.gov/nucleotide/2062851025?report=gbwithparts) | | F: GCCCATACATGACAGCAAGTCT | 116 |
|  |  |  | R: GCACCTTGTCACAGAGCAGATA |  |

**Supplementary Table 4.** Antibody information

| Name of antibody | Dilution ratio | Source | Item number/Batch | Manufacturing company | Headquarters address |
| --- | --- | --- | --- | --- | --- |
| The primary antibodies | | | | | |
| ERK | 1:1000 | Rabbit | #4695 | Cell Signaling Technology, Inc. | Danvers, Massachusetts, USA |
| IκBα | 1:1000 | Rabbit | #9242 | Cell Signaling Technology, Inc. | Danvers, Massachusetts, USA |
| JNK | 1:1000 | Rabbit | #9252 | Cell Signaling Technology, Inc. | Danvers, Massachusetts, USA |
| NOD2 | 1:500 | Rabbit | DF12125 | Affinity Biosciences | Cincinnati, Ohio, USA |
| p38 | 1:1000 | Rabbit | #8690 | Cell Signaling Technology, Inc. | Danvers, Massachusetts, USA |
| p65 | 1:1000 | Rabbit | #8242 | Cell Signaling Technology, Inc. | Danvers, Massachusetts, USA |
| p-ERK | 1:1000 | Rabbit | #4370 | Cell Signaling Technology, Inc. | Danvers, Massachusetts, USA |
| p-IκBα | 1:1000 | Rabbit | #2859 | Cell Signaling Technology, Inc. | Danvers, Massachusetts, USA |
| p-JNK | 1:1000 | Mouse | #9255 | Cell Signaling Technology, Inc. | Danvers, Massachusetts, USA |
| p-p38 | 1:1000 | Rabbit | #4511 | Cell Signaling Technology, Inc. | Danvers, Massachusetts, USA |
| p-p65 | 1:1000 | Rabbit | #3033 | Cell Signaling Technology, Inc. | Danvers, Massachusetts, USA |
| RIPK2 | 1:500 | Rabbit | DF6967 | Affinity Biosciences | Cincinnati, Ohio, USA |
| β-Actin | 1:2000 | Mouse | UM4001 | Utibody | Tianjin, China |
| The secondary antibodies | | | | | |
| Goat anti-Rabbit IgG-HRP | 1:4000 | Goat | S0001 | Affinity Biosciences | Cincinnati, Ohio, USA |
| Goat anti-Mouse IgG-HRP | 1:4000 | Goat | S0002 | Affinity Biosciences | Cincinnati, Ohio, USA |

**Supplementary Figure 1.** The effect of different concentrations of DMSO on the viability of ORECs. ^1^ORECs: ovine ruminal epithelial cells. Cells were treated with different concentrations of DMSO for 24 h. ^2^Results are presented as the Mean±SEM. ^a–d^Values in the same row with different letters are significantly different (*P* <  0.05).

**Supplementary** Figure 2. The effect of different concentrations of GSK 717 on the cytokine content of MDP stimulated ORECs. ^1^ORECs: ovine ruminal epithelial cells; MDP: muramyl dipeptide; GSK717, inhibitor of NOD2 pathway. Cells were treated with different concentrations of GSK717 for 1 h followed by 25 μg/mL MDP for 6 h. (A-B) The levels of IL-6 and IL-8, respectively. ^2^Results are presented as the Mean±SEM. ^a–d^Values in the same row with different letters are significantly different (*P* <  0.05).


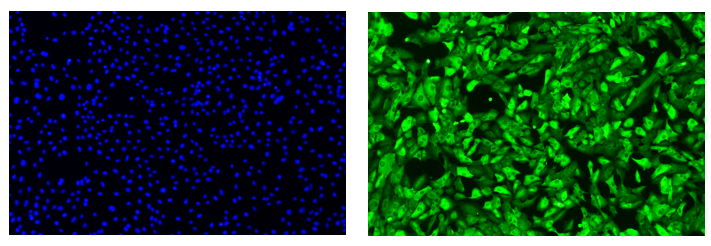


**Supplementary Figure 3.** Results of pan-Cytokeratin Immunofluorescence Identification for ovine ruminal epithelial cells (100**X**). The identification results were provided by Cellverse Co., Ltd. (Shanghai, China).
